# Effects of Alumina Bubble Addition on the Properties of Corundum–Spinel Castables Containing Cr_2_O_3_

**DOI:** 10.3390/ma17133139

**Published:** 2024-06-27

**Authors:** Haonan Chen, Xingfu Shi, Jing Chen, Mengyang Sang, Haoxuan Ma, Xinhong Liu, Quanli Jia

**Affiliations:** 1 School of Materials Science and Engineering, Henan Key Laboratory of High Temperature Functional Ceramics, Zhengzhou University, Zhengzhou 450001, China; chenhaonan2333@163.com (H.C.); chenjing202312@163.com (J.C.); sangmengyang@gs.zzu.edu.cn (M.S.); liuxinhong@zzu.edu.cn (X.L.); 2 Administration for Market Regulation of Xihua County, Xihua, Zhoukou 466600, China; xingfushi1988@outlook.com

**Keywords:** alumina bubbles, corundum–spinel castables, purging plug, box dimension, thermal shock resistance

## Abstract

Purging plugs made of corundum–spinel castables containing Cr_2_O_3_ have been widely utilized in secondary refining process. However, their poor thermal shock resistance has greatly limited the improvement of their service life. Aiming to enhance their properties, we introduced alumina bubbles (ABs) to corundum–spinel castables, and the effects of the AB addition on the properties of the castables are studied in this manuscript. The results indicate that the apparent porosity, permanent linear change, cold strength, and hot strength all increased with an increasing AB amount. The thermal shock resistance of the samples with the AB addition was improved; the residual strength and residual strength ratio of the sample with 4 wt% ABs was the best. The effects of ABs on the tabular alumina aggregate distribution and relationship between the cold strength of the samples and the AB content was evaluated via the box dimension method. With the increments of AB content, the box dimension value of the tabular alumina within the samples significantly decreased, indicating that the tabular alumina aggregate distribution was related to the amount of ABs. In addition, the relationship between the box dimension and the strength was also established.

## 1. Introduction

Purging plugs play a crucial role in secondary refining processes to produce high-quality steel; their functions include facilitating reactions, discharging harmful gases, separating impurities from molten steel, and stirring the molten steel in steel ladles via flowing argon gas [1,2]. Unfortunately, the service life of purging plugs is dramatically shorter in comparison to that of steel ladle linings because of their severe working environment, including thermal stress derived from a larger temperature gradient, corrosion by slag and molten steel, and severe erosion wear by the oxygen cleaning process [3,4,5]. To meet the strict requirements, corundum–spinel castables containing Cr_2_O_3_ have been widely used to fabricate purging plugs due to their impressive slag resistance and excellent hot strength [6], but their poor thermal shock resistance (TSR) results in thermal spalling and fracture, thereby reducing their service life [7]. To respond to these issues, some attempts have been made to improve the TSR of corundum–spinel castables, such as adding zirconia, zirconia–alumina, alumina nanopowders, etc. [8,9,10,11]; however, the TSR of the castables needs to be further enhanced.

Refractory aggregates make up a larger proportion in castables, generally about 70 wt%, which greatly influences their properties. Recently, some works have been conducted to enhance their properties by changing the type and amount of corundum–spinel aggregates in castables. For instance, Tang et al. [12] added porous multi-component CaO-MgO-Al_2_O_3_ aggregates into castables and found that the bonding of interfaces between the aggregates and the matrix was strengthened and that small pores in aggregates can remit thermal stress, resulting in an incensement of the TSR of the specimens. Chen et al. [13] found that the thermal conductivity of corundum–spinel castables was reduced and the TSR was improved by adding porous corundum spheres with a microporous shell and macroporous core. Liu et al. [14] revealed that corundum–spinel castables containing as-formed bonite (CA_6_) aggregates resulted in good TSR, which was due to the released thermal stress and deviated cracks by CA_6_ flakes and their micropores. Liu et al. [15] found that alumina–spinel castables with resin-coated tabular alumina aggregates possessed an improved TSR, which, due to the micro-space formed along the boundary of the aggregates and matrices, can alleviate thermal stress, prolonging the crack propagation path and decelerating the crack propagation rate. Unfortunately, these castables still face some challenges, including a high cost and complex procedures. Hence, selecting a commercial aggregate to cope with industrial requirements should be more feasible.

Alumina bubbles (ABs) have been commonly used to fabricate porous corundum bricks and lightweight thermal insulation refractories due to their excellent performance of lower bulk density, lower apparent porosity, and higher closed porosity [16,17]. For example, Li et al. [18] attempted to introduce ABs into mullite castables and found that the residual strength ratio of the castables could be improved from 63.2% to 88.6%. Herein, ABs were added into corundum–spinel castables, and the effect of the AB content and heat treatment temperature on the physical properties and high-temperature properties of the corundum–spinel castables are studied in this manuscript. The results demonstrate that decrease in the thermal conductivity and the improvement of the TSR of corundum–spinel castables can be obtained by adding ABs, due to the ABs possessing a lower bulk density, a spherical shape, and a hollow structure.

## 2. Experimental

### 2.1. Material Preparation and Characterization

Tabular alumina aggregates (6–3 mm, 3–1 mm, 1–0.5 mm, and 0.5–0 mm, Almatis, Qingdao, China), alumina bubbles (1–0.5 mm, bulk density: 0.95 g/cm^3^, Henan Sicheng Yanmo Co., Zhengzhou, China), fused spinel powder (MgO: 25%, <0.074 mm, Luoyang Lier Co., Luoyang, China), white corundum powder (Al_2_O_3_: 99.0%, <0.088 mm, Kaifeng Tenai Co., Kaifeng, China), ultrafine alumina (Al_2_O_3_ > 99.5%, d_50_ = 1.2 µm, Henan Tianma Co., Zhengzhou, China), chromia powder (Cr_2_O_3_ > 98%, <20 μm, Luoyang Zhengjie Co., Luoyang, China), and calcium aluminate cement (Secar 71, Imerys, Tianjin, China) were used as raw materials.

The mass ratio of the aggregates (25% 6–3 mm, 25% 3–1 mm, 10% 1–0.5 mm, and 10% 0.5–0 mm) and the matrix was 70:30 in the castables. The 1–0.5 mm tabular alumina aggregates were replaced by ABs, at amounts of 0 wt% (0 Vol%), 2 wt% (7 Vol%), 4 wt% (13.3 Vol%) and 6 wt% (19.0 Vol%). The specimens were coded as AB0, AB2, AB4, and AB6, respectively. Firstly, the aggregates and matrices were uniformly mixed and blended with water, and then the mixtures were cast into 25 mm × 25 mm × 150 mm bars. Subsequently, they were demolded after aging in ambient air for 24 h, and dried at 110 °C for 24 h. Finally, they were calcined at 1100 °C, 1400 °C, and 1600 °C for 3 h, respectively.

The physical properties of the castables, including the apparent porosity (AP), bulk density (BD), permanent linear change (PLC), cold modulus of rupture (CMOR), and cold crushing strength (CCS) were carried out. The AP and BD were identified according to GB/T2997-2015. The PLC was evaluated according to GB/T5988-2022. The CMOR and CCS were measured at room temperature according to GB/T3001-2017 and GB/T5072-2008, respectively. The hot modulus of rupture (HMOR) was identified according to GB/T3002-2017 at 1400 °C for 0.5 h via the 3-point bending method. In addition, the TSR of the castables after heating at 1600 °C was evaluated at 1100 °C for 0.5 h using the water quenching method, and the residual CMOR (CMORst) and retained strength ratios (RSR) were measured.

The microstructures of the fracture specimens and the polished cross-sections (embedded in the resin) of the samples and ABs were characterized by a scanning electron microscope (SEM, TESCAN MIRA LMS, Brno, Czech Republic) equipped with an energy-dispersive spectrometer (EDS, Oxford Xplore, Oxford, UK). The phase composition of the specimens was examined by X-ray diffraction (XRD, Philips X′Pert, Almelo, The Netherlands) with Cu Kα radiation (λ = 1.5406 Å) at 40 kV, 40 mA, and a scan range of 10°−80° at 10°/min.

### 2.2. Fractal Method

Fractal theory, as a quantitative approach, directly reveals the inherent laws within complex and irregular systems, such as irregular and coarse geometric shapes in nature [19,20]. Hence, fractal theory has diverse applications in numerous disciplines, notably in the realm of material science. Its utility spans the characterization of pores in porous materials [21,22,23], cracks [24,25], and other morphology analyses. The box-counting dimension method is a prevalent technique for calculating the box dimension (*D_b_*) of structures, facilitating the computation of dimensions for complex structures or shapes.

Let *F* be any bounded non-empty subset of *R^n^*, where *N_δ_*(*F*) represents the minimum number of boxes that are required to encompass *F* with the box edge length *δ* (*δ* > 0). The relationship between *N_δ_*(*F*) and *δ* can be defined as Equation (1) [26,27]:

(1) $$N_{\delta }(F)\propto \delta^{-D_{b}}$$

where *D_b_* is the box dimension. The opposite number of the limit of ln*N_δ_*(*F*)/ln*δ* as δ approaches 0 is *D_b_*. By selecting the *δ* of different scales, the continuous point graph of (ln*N_δ_*(*F*), ln*δ*) can be obtained. At a small scale, ln*N_δ_*(*F*) and ln*δ* are linearly dependent, and Equation (1) can be equivalent to Equation (2):

(2) $$D_{b}\approx -\frac{d\ln N_{\delta }\left(F\right)}{d\ln\delta }$$

Therefore, the negative of the slope of the line obtained from linear regression on the scatter plot is denoted as *D_b_*.

In this work, the cross-sections of the castables fired at 1600 °C were selected and recorded by a digital camera. Image processing software was utilized to capture high-resolution images of the samples, and the tabular alumina aggregates and matrices were distinguished by highlighting them in white and black, respectively.

## 3. Results and Discussion

### 3.1. Characterization of Alumina Bubbles

**Figure 1:**
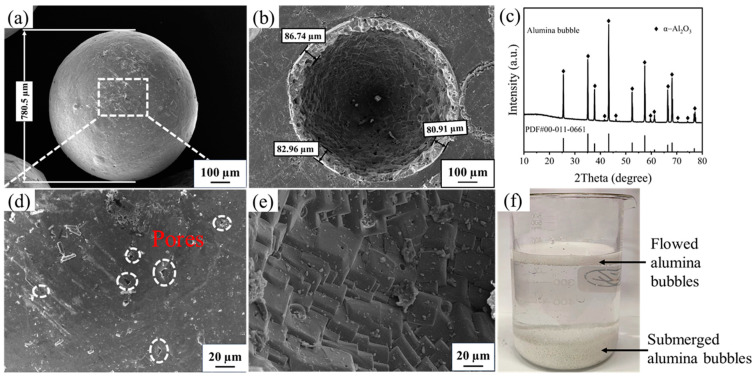
(**a**,**d**) surficial morphology of ABs, (**b**,**e**) internal morphology of ABs, (**c**) XRD pattern of ABs, and (**f**) floating state of ABs in water.

Figure 1 shows the microstructures of the ABs. As shown in Figure 1a–c, the AB particles were hollow and spherical, the shell thickness of the ABs was about 83.5 µm, and small pores were observed on their surface. The internal morphologies of the ABs are shown in Figure 1d; it can be observed that the shells of the ABs were dense and shaped by stacking cubic alumina crystals. Their phase composition was corundum (ICDD No. 00-011-0661) (Figure 1e). As shown in Figure 1f, some ABs floated on the surface of water, whereas others sank to the bottom, suggesting that a certain number of the ABs were broken.

### 3.2. Physical Properties of the Castables

**Figure 2:**
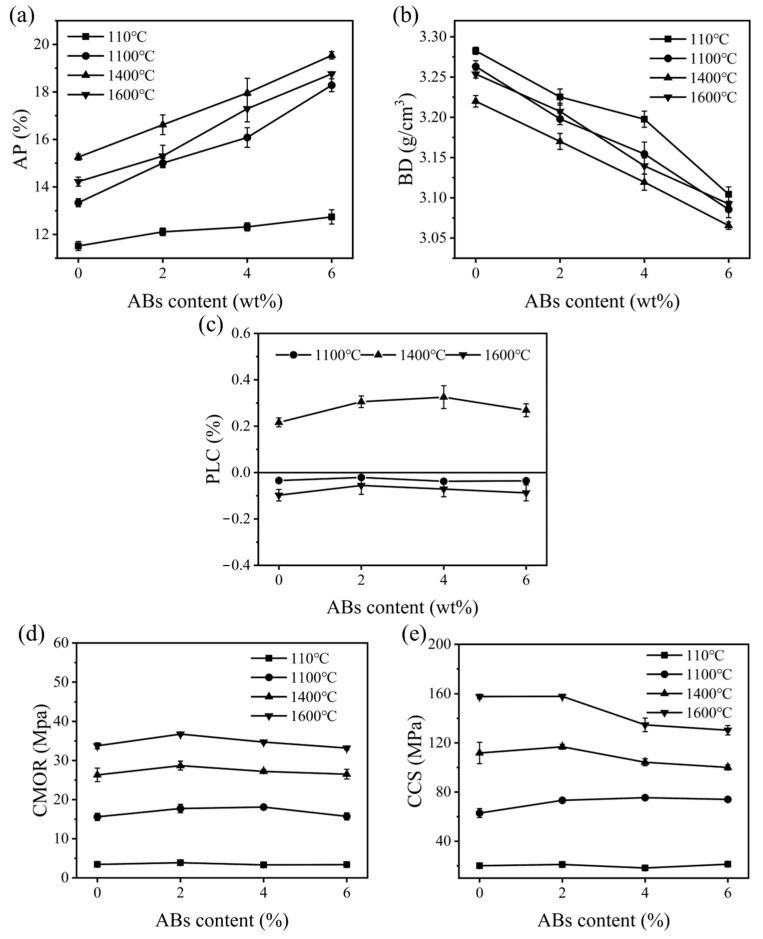
Physical properties of the samples. (**a**) AP, (**b**) BD, (**c**) PLC, (**d**) CMOR, and (**e**) CCS.

Figure 2 presents the physical properties of the samples treated at different temperatures. As shown in Figure 2a,b, as the ABs increased, the AP values of the castables after heat-treating at 110 °C, 1100 °C, 1400 °C, and 1600 °C increased from 11.5% to 12.7%, from 13.3% to 18.3%, from 15.3% to 19.5%, and from 13.6% to 18.8%, respectively, which may have been due to the presence of surface pores on the ABs. Additionally, the hollow structure of the ABs was partially destroyed during the mixing process, thereby increasing the apparent porosity. In contrast, the BD exhibited a corresponding downward trend, decreasing from 3.28 g/cm^3^ to 3.10 g/cm^3^, from 3.26 g/cm^3^ to 3.09 g/cm^3^, from 3.22 g/cm^3^ to 3.07 g/cm^3^, and from 3.28 g/cm^3^ to 3.09 g/cm^3^, which may have been because the BD of the ABs was significantly lower than that of the tabular alumina aggregates. On the other hand, Figure 2a,b indicate that with the treatment temperature increasing from 110 °C to 1600 °C, the AP values of sample AB2 were 12.1%, 15.0%, 16.6%, and 15.3%. As shown in Figure 2c, the PLC values of specimens were slightly changed with an increasing AB content from 0% to 6%. The CMOR and CCS values of the samples are shown in Figure 2d,e. As revealed in Figure 2d,e, the CMOR values increased to the maximums of 3.87 MPa, 18.10 MPa, 28.70 MPa, and 36.76 MPa, respectively, and decreased afterward. The CCS value exhibited a similar trend, which was 21.1 MPa, 75.4 MPa, 116.8 MPa, and 157.9 MPa, respectively. Overall, these results demonstrate that the physical properties of the castables can be improved by introducing a certain amount of ABs, which is similar to a previous report [28].

### 3.3. Thermo-Mechanical Properties of the Castables

**Figure 3:**
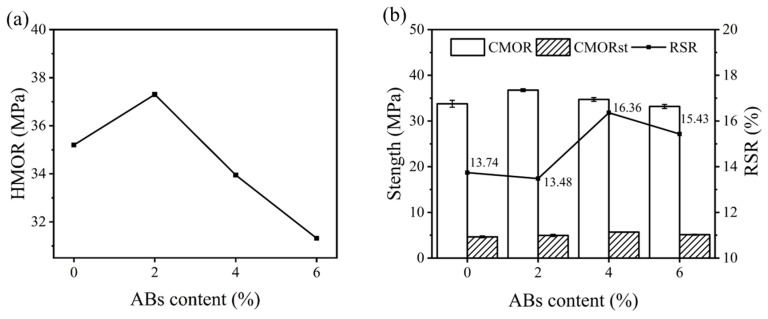
(**a**) HMOR and (**b**) TSR of the samples with different amounts of ABs.

Figure 3 shows the HMOR and TSR of the specimens. As presented in Figure 3a, with increasing ABs, the HMOR value was 35.2 MPa, 37.3 MPa, 34.0 MPa, and 31.3 MPa for samples AB0, AB2, AB4, and AB6, respectively, exhibiting a similar trend to their cold strength. Figure 3b shows the correlation between the CMORst and the RSR of the castables. It can be seen that the CMORst value slightly increased from 4.64 MPa to 5.68 MPa by increasing the ABs content from 0% to 4%. The RSR values of samples AB0-AB6 were 13.74%, 13.48%, 16.36%, and 15.43%, respectively. It could be inferred that the TSR of the samples could be slightly improved by adding ABs, which was attributed to the hollow structure of the ABs buffering the thermal stress during thermal shock. Additionally, the decrease in strength led to an improvement in ductility.

### 3.4. Aggregate Distribution of the Castables

**Figure 4:**
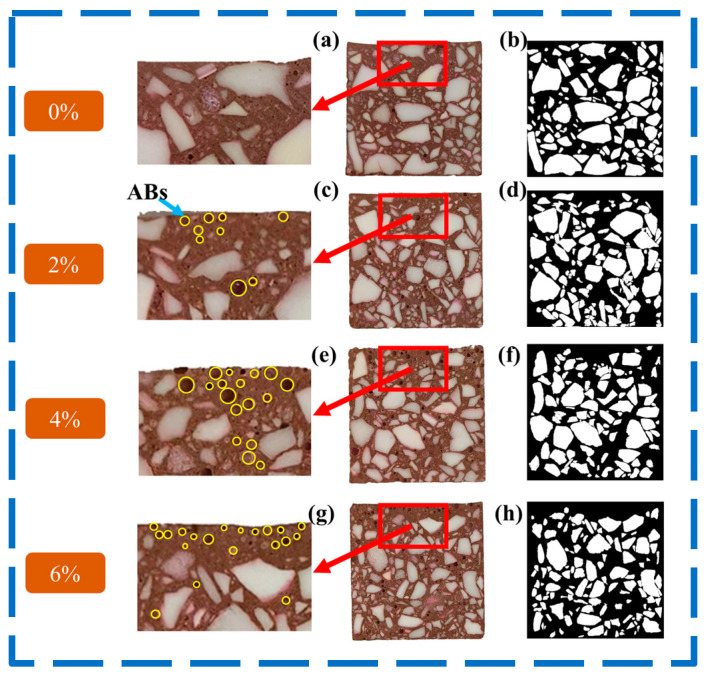
Digital photos of the cross-sections and binarized pictures of the samples: (**a**,**b**) AB0, (**c**,**d**) AB2, (**e**,**f**) AB4, and (**g**,**h**) AB6. (Here, yellow circles refer to ABs).

**Figure 5:**
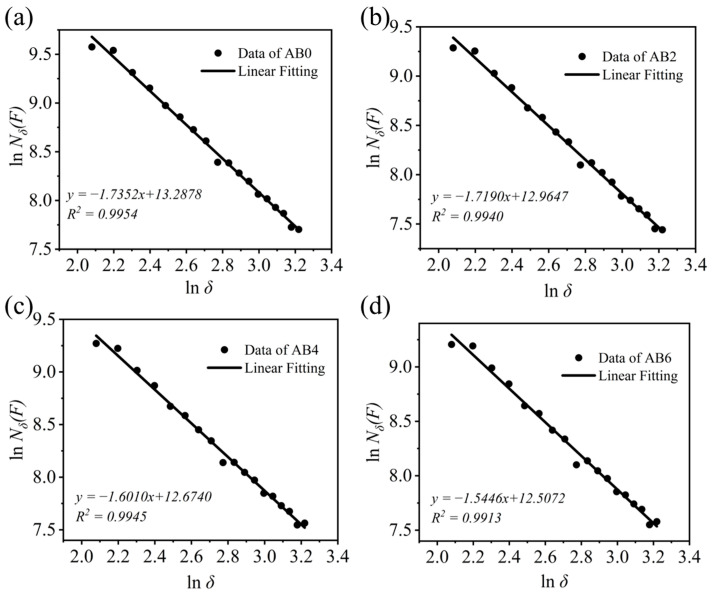
The equations of linear regression of the samples: (**a**)AB0, (**b**) AB2, (**c**) AB4, and (**d**) AB6.

Figure 4 shows the digital photos and their binary black and white images of the castables. As shown in Figure 4, the ABs (marked with a yellow circle) were occupied the top area of the castables, and with the increasing ABs, the distribution of the tabular alumina aggregates tended to be more uneven. The results of the *D_b_* of the samples with different AB contents are shown in Figure 5. The slope of the regression lines for samples AB0-AB6 was 1.7352, 1.7190, 1.6010, and 1.5446, respectively. This indicates that the *D_b_* values of the tabular alumina aggregates decreased with an increase in the AB content. The study conducted by He et al. [29] showed that the *D_b_* correlated with the uniformity of the distribution of the second-phase components in the sample, where a larger *D_b_* indicated a more uniform distribution of the particles. In the two-dimensional situation, the maximum value of the *D_b_* was 2. Consequently, the addition of ABs to the castables resulted in an irregular distribution of the tabular alumina aggregates in the castables. Moreover, the uneven distribution of the tabular alumina aggregates intensified with the increasing amount of ABs in the samples. The uneven distribution of the aggregates was due to the tendency of the low-density ABs floating to the top of the samples during the vibration process, making the tabular alumina aggregates migrated upward within the sample, which can be evidently seen in Figure 4.

**Figure 6:**
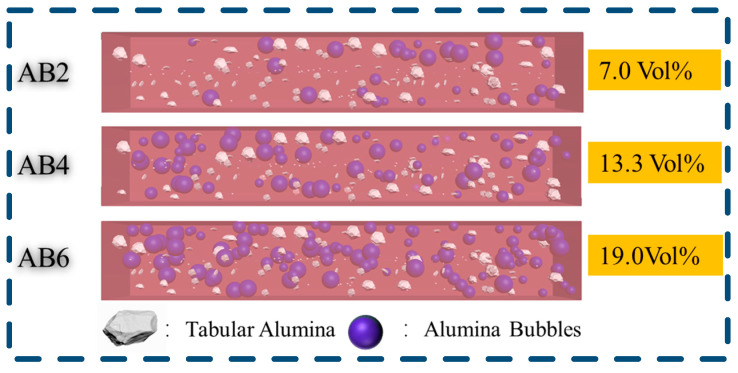
Schematic diagram of the volume content of ABs in castables.

**Figure 7:**
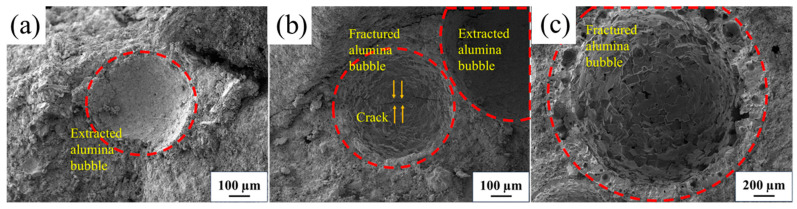
The fracture morphologies of AB2 fired at (**a**) 1100 °C, (**b**) 1400 °C, and (**c**) 1600 °C.

Figure 6 illustrates the volume ratio of the ABs in the samples. As presented in Figure 7, the volumes of the ABs were 7.0%, 13.3%, and 19.0%, respectively for sample AB2, sample AB4 and sample AB6, revealing that a significant change in the volume fraction was generated by adding lightweight ABs. Therefore, the change in the volume percentage may be resulted in an uneven distribution of the tabular alumina aggregates in the castables, which triggered a decrease in the mechanical properties of the samples. As for the samples with ABs, with the decreased *D_b_*, the CMOR, CCS, and HMOR values were decreased as well, which may be ascribed to the uneven distribution of the tabular alumina aggregates, resulting in an uneven stress distribution and thereby decreasing their cold and hot strength.

### 3.5. Discussion

The microstructures of fracture sample AB2 fired at different temperatures are exhibited in Figure 7. As shown in Figure 7a, it can be seen that a majority of the ABs were extracted from the matrix in the sample after firing at 1100 °C, leaving behind spherical pits and suggesting that their strength was relatively lower, which is shown in Figure 2d. The factual ABs were seen in the sample fired at 1400 °C, and some cracks were propagated through the intergranular ABs (Figure 7b), indicating that the cold strength of the samples was noticeably enhanced. As shown in Figure 7c, after firing at 1600 °C, the ABs grains exhibited strong bonding with the matrix, and crack propagation primarily occurred through the AB grains. These results demonstrate that with increases in the firing temperature from 1100 °C to 1600 °C, the interfacial bonding between the ABs and the matrices was progressively strengthened. The fracture types containing intergranular fracture and transgranular fracture of the ABs reveal that the increases in the firing temperature enhanced the interfacial bonding between the ABs and the matrix, thereby improving the cold strength of the castable.

The physical properties, HMOR, and TSR of the samples were noticeably affected by adding ABs. The AP values were increased, which may be ascribed to the hollow structure of the ABs being broken during the stirring process and some pores distributed on the shells of the ABs. The PLC values of the samples were slightly changed. The CMOR, CCS, and HMOR values of the castables were greatly impacted by introducing ABs. When the AB amount was 2 wt%, their strength was greater than that of the samples without ABs; this was owing to the spherical structure of the ABs facilitated stress buffering. With increases in the ABs from 2% to 6%, their strength decreased, which may be due to the volume fraction of the ABs increasing and the matrix fraction decreasing correspondingly, thereby decreasing the bonding mode of the ABs, tabular alumina aggregates, and the matrix. Hence, the non-uniform arrangement of the aggregates led to an inhomogeneous distribution of the strength and uneven stress distribution, consequently causing a reduction in strength [30], which is beneficial to improving the thermal shock resistance of castables with AB addition.

**Figure 8:**
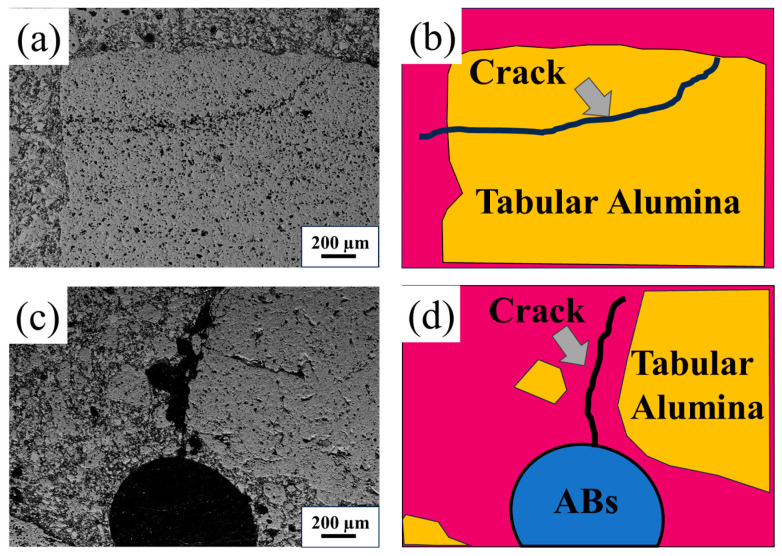
SEM photos and schematic diagrams of crack propagation in the samples: (**a**,**b**) AB0 and (**c**,**d**) AB4.

The SEM photos and schematic graphs of sample AB0 and sample AB4 after thermal shock are displayed in Figure 8. A trans-granular crack can be seen in sample AB0, indicating that higher thermal stress was formed after thermal shock, and therefore, the TSR of the sample without AB addition was the worst [15], which may be ascribed to their dense structure and higher strength. As for sample AB4, the propagation of the crack was impeded by the ABs (Figure 8c,d), resulting in a prolonged path for crack propagation and a reduction in the rate of crack propagation. This effect may be attributed to the hollow structure of ABs, which can effectively buffer thermal shock energy, and their spherical shape can alleviate stress concentration, thereby enhancing the thermal shock resistance of the specimens.

## 4. Conclusions

In this manuscript, the effects of AB addition on the properties of castables was investigated. With increasing of AB content, the apparent porosity of the castables increased and the bulk density decreased. The cold strength and hot strength firstly increased when the AB addition was 2%, and then decreased with further increasing of the Abs amount, which may be ascribed to the addition of ABs resulted in a heterogeneous distribution of the tabular alumina aggregates and finally led to the decrease in strength of the samples. The thermal shock resistance of the sample with 4% ABs was noticeably increased, which can be ascribed to the hollow structure and spherical shape of the ABs, which prolonged the crack propagation path and reduced the formation of cracks suffering from thermal shock, thereby increasing the thermal shock resistance of the castables.

## Data Availability

Data are contained within the article.
